# The extra cost of comorbidity: multiple illnesses and the economic burden of non-communicable diseases

**DOI:** 10.1186/s12916-017-0978-2

**Published:** 2017-12-08

**Authors:** Sébastien Cortaredona, Bruno Ventelou

**Affiliations:** 1 Aix-Marseille University, INSERM, IRD, SESSTIM, Sciences Economiques & Sociales de la Santé & Traitement de l’Information Médicale, 19-21 boulevard Jean Moulin, 13005 Marseille, France; 2 ORS PACA, Observatoire régional de la santé Provence-Alpes-Côte d’Azur, Marseille, France; 3 Aix-Marseille Univ., CNRS, EHESS, Centrale Marseille, Aix-Marseille School of Economics, Marseille, France

**Keywords:** Cost of illness, Comorbidity, Chronic diseases, Prevention policies

## Abstract

**Background:**

The literature offers competing estimates of disease costs, with each study having its own data and methods. In 2007, the Dutch Center for Public Health Forecasting of the National Institute for Public Health and the Environment provided guidelines that can be used to set up cost-of-illness (COI) studies, emphasising that most COI analyses have trouble accounting for comorbidity in their cost estimations. When a patient has more than one chronic condition, the conditions may interact such that the patient’s healthcare costs are greater than the sum of the costs for the individual diseases. The main objective of this work was to estimate the costs of 10 non-communicable diseases when their co-occurrence is acknowledged and properly assessed.

**Methods:**

The French Echantillon Généraliste de Bénéficiaires (EGB) database was used to assign all healthcare expenses for a representative sample of the population covered by the National Health Insurance. COIs were estimated in a bottom-up approach, through regressions on individuals’ healthcare expenditure. Two-way interactions between the 10 chronic disease variables were included in the expenditure model to account for possible effect modification in the presence of comorbidity(ies).

**Results:**

The costs of the 10 selected chronic diseases were substantially higher for individuals with comorbidity, demonstrating the pattern of super-additive costs in cases of diseases interaction. For instance, the cost associated with diabetes for people without comorbidity was estimated at 1776 €, whereas this was 2634 € for people with heart disease as a comorbidity. Overall, we detected 41 cases of super-additivity over 45 possible comorbidities. When simulating a preventive action on diabetes, our results showed that significant monetary savings could be achieved not only for diabetes itself, but also for the chronic diseases frequently associated with diabetes.

**Conclusions:**

When comorbidity exists and where super-additivity is involved, a given preventive policy leads to greater monetary savings than the costs associated with the single diagnosis, meaning that the returns from the action are generally underestimated.

**Electronic supplementary material:**

The online version of this article (doi:10.1186/s12916-017-0978-2) contains supplementary material, which is available to authorized users.

## Background

The Organisation for Economic Co-operation and Development [1] predicts that healthcare expenditure will continue to rise, putting pressure on public budgets over the next decades. European countries are probably the most exposed to this risk given the factors of population aging and comprehensive public health insurance coverage. However, Europe has no coherent (i.e. using consistent concepts and methods) or comprehensive empirically based framework that can provide measures and forecasts of the burden of healthcare in a disease-oriented approach. As a result, policy-makers are confronted with competing estimates of healthcare costs for particular diseases or risk factors, with each study having its own data and methods [2–7].

In 2007, the Dutch Center for Public Health Forecasting of the National Institute for Public Health and the Environment summarised the methodology for general cost-of-illness (COI) studies and provided guidelines that can be used to set up such studies [8]. They emphasised the fact that a common problem in COI analyses is how to deal with patients’ comorbidities. Top-down analyses, in which costs for a given disease are calculated by multiplying aggregate health expenditure by the suspected proportion of the ‘top’ amount spent on that disease, require costs to be attributed to a single diagnosis. Thus, comorbidity is basically not taken into account. On the other hand, a bottom-up approach, in which each unit of healthcare used on a patient is attributed to a disease, still has trouble accounting for comorbidity. A classic example is a consultation for diabetes, which is also a major risk factor for cardiovascular disease. In an ideal bottom-up approach, the costs of this consultation should be applied both to heart disease and to diabetes, with – still ideally – the relative shares reflecting the importance of the consultation in the treatment of each disease, which becomes rapidly unattainable.

We therefore aimed to develop a comprehensive ‘bottom-up’ approach using person-level data to estimate the costs of chronic diseases using a medico-administrative database. One of the main strengths of our approach is that it takes into account the comorbidity issue, a key factor with the older population. Recent strategies using regression-based frameworks [9–11], have also been developed to account for excess spending caused by the presence of comorbidities. Compared to top-down approaches, these types of person-level costing may produce more valid estimates in patients with multiple chronic diseases, as expenditures for comorbidities and complications are better captured [9].

For this study, we focused on 10 chronic disease groups, namely heart disease, stroke, diabetes, cancers (with a focus on breast, liver, lung, colorectal, stomach, oesophageal, kidney and pancreatic cancers), alcohol use disorders, cirrhosis, neurological disorders, major depression, respiratory illness (chronic obstructive pulmonary disease, asthma), and chronic kidney disease (CKD). Our cost-calculation methods address the possible coexistence of these 10 chronic diseases within the same subject, which may interact in the selection of treatments, potentially making costs of diseases ‘super-additive’.

A ‘simulation exercise’ estimates the cost savings by a health system upon elimination of one disease, e.g. diabetes. Where there is super-additivity, there are far greater cost savings than with simply additive costs, meaning that the calculations performed to estimate the benefits (returns) of preventive action generally underestimate them.

## Methods

### Study population

We used the Echantillon Généraliste des Bénéficiaires (EGB) database, a permanent, representative and anonymised sample of people affiliated with the three major National Health Insurance funds [12]. These funds cover more than 90% of the French general population, divided into salaried workers, agricultural workers and farmers, and self-employed workers. The EGB was created in 2005 by a national random sampling of 1/97th of the French population, stratified for age and sex; it records information on their healthcare consumption and includes data on reimbursement claims for drugs purchased in the community, classified according to the Anatomical Therapeutic Chemical (ATC) index. The EGB is a dynamic cohort, where every 3 months, registered births and foreign immigrants taking up employment in France and their eligible dependents are added to the sample. Conversely, deaths and people withdrawing from the insurance funds covered by the EGB are extracted from the sample [12].

For this study, we selected people aged 18 or above and listed in the EGB on January 1, 2014, and monitored them until the end of 2014. Those who died or withdrew from the insurance funds covered by the EGB during the monitoring period were excluded from the analysis.

### Identification of people with chronic diseases

The list of the 10 chronic diseases selected for this study, with assigned International Classification of Disease (ICD version 10) codes, is provided in Table 1.

**Table 1:** The 10 chronic diseases and assigned International Classification of Disease version 10 (ICD-10) codes

Disease group	Disease	ICM-10 codes
Stroke	Acute haemorrhagic stroke	I60 Subarachnoid haemorrhage
		I61 Intracerebral haemorrhage
	Acute ischemic stroke	I63 Cerebral infarction
	Chronic stroke (any type)	I69 Sequelae of cerebrovascular disease
Heart disease	Acute myocardial infarction	I21 Acute myocardial infarction
		I22 Subsequent myocardial infarction
	Chronic ischaemic heart disease	I20 Angina pectoris
		I23 Certain current complications following acute myocardial infarction
		I25 Chronic ischaemic heart disease
Cancer	Stomach	C16 Malignant neoplasm of stomach
	Colorectal	C18 Malignant neoplasm of colon
		C19 Malignant neoplasm of rectosigmoid junction
		C20 Malignant neoplasm of rectum
		C21 Malignant neoplasm of anus and anal canal
	Lung	C33 Malignant neoplasm of trachea
		C34 Malignant neoplasm of bronchus and lung
	Liver	C22 Malignant neoplasm of liver and intrahepatic bile ducts
	Breast	C50 Malignant neoplasm of breast
	Oesophageal	C15 Malignant neoplasm of oesophagus
	Kidney	C64 Malignant neoplasm of kidney, except renal pelvis
	Pancreatic	C25 Malignant neoplasm of pancreas
Diabetes		E10–E14 Diabetes mellitus
Chronic kidney disease		N18 Chronic kidney disease
Respiratory illness		J41 Simple and mucopurulent chronic bronchitis
		J42 Unspecified chronic bronchitis
		J43 Emphysema
		J44 Other chronic obstructive pulmonary disease
		J45 Asthma
		J47 Bronchiectasis
Cirrhosis		I85 Oesophageal varices
		K70–K77 Diseases of liver
Alcohol use disorders		F10 Alcohol related disorders
Depression		F32 Depressive episode
		F33 Recurrent depressive disorder
Neurological disorders		F00–F03, G30–G31

This study was performed as part of the FRESHER research consortium (FoResight and Modelling for European Health policy and Regulation). We selected non-communicable diseases that contribute to the bulk of deaths worldwide, namely cardiovascular diseases, cancers, diabetes, and chronic lung disease [13]. To these, we added four other health conditions, namely (1) depression, due to its co-morbid status with all of the chronic diseases outlined above, (2) chronic kidney disease, (3) alcohol-related diseases, and (4) chronic neurological disorders, due to their increasing importance in ageing societies. Most of these conditions share commonalities in pathogenesis, aetiology, and risk factors, which are potentially modifiable. With no intervention, their cumulative financial burden is projected to greatly increase in the next decades [14].

Administrative databases, such as drug prescription data, have frequently been used to identify people with chronic diseases in prevalence estimates [15] or epidemiological studies for comorbidity adjustments [16]. In the EGB database, three types of data can be used to identify patients with chronic diseases.

In 2013, Huber et al. [17] developed an updated approach with a special focus on the unambiguous assignment of drug prescriptions to chronic diseases. For chronic diseases, they only included ATC codes, which are exclusively used for the treatment of specific diseases. From 2009 to 2014, we considered that a person had a chronic disease if they had been dispensed at least three drugs in the corresponding ATC category at different times over the calendar year (this threshold of three dispensations was used on various French studies on diabetes [18] and other chronic diseases [19]).

Since 2006, data from the French Medical Information System (Programme de Médicalisation des Systèmes d'information), which covers all French public and private hospitals except military and psychiatric hospitals, have been available for all individuals included in the EGB. In France, each hospital stay is registered in a hospital discharge database from the French Medical Information System and diagnoses are coded using the International Classification of Diseases (ICD-10 [20]) codification either as a primary, related, or significant associated diagnosis. Cchronic diseases were identified using these diagnosis data from 2009 to 2014.

We used long-term illness (LTI) status data from the French social insurance system to identify people with chronic diseases. LTI status is granted to people with long-term and costly diseases, exempting them from co-payments for any associated medical treatment [21]. As with hospital discharge data, chronic diseases were identified from 2009 to 2014 using the ICD-10 classification.

These three sources of data (drug dispensing, hospital discharge and LTI) are available in the EGB database and linked with a unique individual identifier. They were combined to identify prevalent and incident cases for our 10 chronic disease groups.

### Healthcare expenditure data

We used reimbursement data to calculate, for each individual in the database, an aggregate healthcare expenditure in 2014, including primary care and consultations with specialists, (reimbursed) medicines, medical procedures, biological tests, medical devices, emergency care, and hospital inpatient care. This pricing of ambulatory care also takes into account possible co-payment from the patient, except for over-the-counter drugs which are not available in the EGB database. Regarding the hospital sector, this cost-evaluation only takes into account the part of the cost which is reimbursed to hospitals through the diagnosis-related group payment system (through which we can clearly assign a diagnosis using the reason of admission). Diagnosis-related group rates were used as proxies of case costs for public and private not‐for‐profit hospital stays. Some specific costs supported by the hospitals are not included in our modelling, e.g.: costs of clinical research; innovative drugs, etc.

### Estimating the cost of chronic diseases

In a bottom-up design, healthcare consumption is collected at individual patient level and illness costs are modelled at the same level. Compared to a top-down approach, in which total expenditure for a given area or policy is divided by the number of patients with a given disease, the bottom-up approach provides greater accuracy [22]. However, in the French healthcare system, healthcare expenditure on patients, particularly in the ambulatory care system, cannot be directly linked to one specific diagnosis. To overcome this limitation, we chose to estimate the cost associated with each chronic disease using regression models. The marginal costs associated with one disease were estimated on individual-level data as the mean marginal difference in the predicted outcome (total individual healthcare expenditure in 2014) with the chronic disease independent variables switched on or off. This makes it possible to estimate the ‘counterfactual’ of what the cost would have been in the absence of chronic disease, while leaving the other model parameters unchanged. This approach is commonly used to estimate incremental costs for diseases and risk factors [23–25]. 

A useful modelling framework in such cases is a two-stage model [26]. Two-stage models are appropriate for analysing zero-inflated cost data with skewness [27], which is typical in medical expenditure data [26]. Our dataset included both many people with zero healthcare spending and some with extremely high spending. In our two-stage model, the first part *P* (cost > 0) was modelled using a logit model; then, conditional on the healthcare spending being positive, the value of the spending was modelled using gamma regressions with log link [28, 29].

Models were stratified by sex and were age adjusted. The 10 binary chronic disease variables were added as main interest variables. Two-way interactions between these variables were also included in the model to account for possible effect modification in the presence of comorbidities (see latter for a precise assessment of the sign of the modification, namely super-additivity or under-additivity). In order to control for the presence of a chronic condition outside of the list of the 10 selected chronic conditions, we also added ‘other LTI’ as a systematic covariate in the model (at least one ongoing LTI for another chronic condition in 2014).

In each stratum (combination of age categories and sex), the increase in healthcare expenditure attributable to each disease was calculated by subtracting average predicted expenditure for sick people in each category from average predicted expenditure for the individuals with the other disease variables set to 0 (or 1 in case of comorbidity for a given other disease). For example, in given strata i, the cost associated with chronic disease d_j_ (with no other comorbidity) was estimated as follows:

1 $$ \widehat{\mathit{\cos}{t}_{i{d}_j}}=\left[{\widehat{c}}_{i\mid {d}_j=1,{d}_{k_{k\ne j}}=0,\kern0.5em {d_{kl}}_{k,l\ne j}=0\ast 0,{d_{jk}}_{k\ne j}=1\ast 0}\right]-\left[{\widehat{c}}_{i\mid {d}_j=0,{d}_{k_{k\ne j}}=0,\kern0.5em {d_{kl}}_{k,l\ne j}=0\ast 0,{d_{jk}}_{k\ne j}=0\ast 0}\right] $$

Where $\widehat{\mathit{\cos}{t}_{i{d}_j}}$ is cost in 2014 associated with chronic disease j in strata i (no other comorbidity), ĉ_i_ is predicted outcome in strata i, d_i_ is binary chronic disease variable for disease i, and d_jk_ is interaction variable between chronic disease variables d_j_ and d_k_.

We obtained an average cost per capita attributable to each chronic disease by calculating a weighted mean over all strata, with:

2 $$ \overline{\widehat{\mathit{\cos}{t}_{d_j}}}=\frac{1}{N}{\sum}_{i=1}^N{w}_i\ast \kern0.5em \widehat{\mathit{\cos}{t}_{i{d}_j}} $$

Where $\overline{\widehat{\mathit{\cos}{t}_{d_j}}}$ is COI calculation for disease d_j_, N is total number of strata (=combination of sex and age categories), and w_i_ is weight for stratum i (=number of individuals in the sample of persons with at least one disease).

### Estimating super-additivity in costs, aggregated data and the simulation exercise

The econometric models allowed estimation of (1) simple COI calculations (10 diseases with no other comorbidity; $\overline{\widehat{\mathit{\cos}{t}_{d_j}}}$), and (2) coupled COI calculations for the 45 two-by-two possible comorbidities ((10 × 9)/2), $\overline{\widehat{\mathit{\cos}{t}_{\left.{d}_j\right|{d}_k}}}$ for the cost of disease d_j_ in the presence of disease d_k_ (see Additional file 1 for a reformulation of equation (1) in case of comorbidity). The precise assessment of ‘super-additivity’ was then computed on the basis of the following comparison:

3 $$ \overline{\widehat{\mathit{\cos}{t}_{\left.{d}_j\right|{d}_k}}}+\overline{\widehat{\mathit{\cos}{t}_{\left.{d}_k\right|{d}_j}}}<>\overline{\widehat{\mathit{\cos}{t}_{d_j}}}+\overline{\widehat{\mathit{\cos}{t}_{d_k}}} $$

When the left-hand side of the inequality was superior to the right-hand side, the assessment was ‘super-additivity’ – the two estimated costs coupled in comorbidity were superior to the addition of the two separate COI calculations with no other comorbidity. Aggregate estimates were determined by multiplying the per capita estimates by the number of people in the corresponding strata. Since the EGB was created by a national random sampling of 1/97th of the French population, we multiplied all figures by 97 in order to get an estimate of the national expenditure associated with each chronic disease.

We also performed a simulation exercise in which we ‘eliminated’ diabetes from the two-stage models, by switching the dummy variable to ‘off’ in all cases (the computer-program was able to estimate the virtual elimination of the nine other diseases; estimates are available on request). The population remained the same; we only simulated a ‘virtual’ situation of people suddenly healed of the disease. The objective of this simulation was to estimate how the savings resulting from preventive action on one disease would also affect the treatment costs of the nine remaining chronic diseases through its super-additivity effect. Confidence intervals (CIs) were computed via Monte Carlo simulations (10,000 replications). Statistical analyses were performed with SAS statistical software, version 9.4 (SAS Institute Inc., Cary, NC, USA).

## Results

### Cohort characteristics

Of the 476,252 people included in the cohort, 48.9% were men and 22.7% were aged over 65, which is relatively close to the census estimates for the French general population in 2014 (23.1% [30]; Table 2).

**Table 2:** Sociodemographic characteristics of the study population (*n* = 476,252)

	%
Sex	
Male	48.92
Female	51.08
Age^a^	
18–39	34.78
40–49	18.40
50–59	16.61
60–64	7.50
65–69	6.73
70–74	4.45
75–79	4.08
80–84	3.57
85–89	2.36
>89	1.53

^a^Age group definition was based on the overall age-specific prevalence of the 10 selected chronic diseases; broader age groups (10/20 years) were used for individuals under 60 in order to obtain sufficiently sized age groups

### Prevalence of chronic diseases

Using LTI, drug dispensing and hospital discharge data, we found that the chronic disease with the highest number of cases was respiratory illness (7.8%), followed by diabetes (7.1%) and heart disease (3.7%) (Table 3). Prevalence rates tended to be higher among men. Prevalence estimates according to the identification method are available in Table 3.

**Table 3:** Prevalence estimates according to the identification method (*n* = 476,252)

	Long-Term Illness data^a^		Hospital discharge data^b^		Drug Dispensing data^c^		Overall prevalence estimates	
	*N*	%	*N*	%	*N*	%	*N*	%
Stroke	1938	0.4	3077	0.7			4348	0.9
Heart disease	11,764	2.5	12,461	2.6			17,465	3.7
Cancer^d^	10,482	2.2	7423	1.6			12,656	2.7
Diabetes	24,705	5.2	16,245	3.4	30,454	6.4	33,686	7.1
Chronic kidney disease	1253	0.3	4761	1.0			5267	1.1
Respiratory illness^e^	1081	0.2	6209	1.3	34,671	7.3	37,203	7.8
Alcohol use disorder	927	0.2	6188	1.3			6683	1.4
Cirrhosis	1073	0.2	3847	0.8			4360	0.9
Major depression	9168	1.9					9168	1.9
Neurological disorders	3355	0.7	4201	0.9	1112	0.2	5945	1.3

^a^Chronic diseases were identified using the ICD-10 classification (Table 1) of the Long-Term Illness registry

^b^Chronic diseases were identified using the ICD-10 classification (Table 1) of diagnoses reported in the hospital discharge database

^c^We considered that a person had a chronic disease if they had been dispensed at least three drugs in the corresponding ATC category [17] at different times over the calendar year

^d^Breast/Lung/Colorectal/Stomach/Liver/Kidney/Pancreatic/Oesophageal

^e^Chronic obstructive pulmonary disease, asthma

### Prevalence of comorbidities

Of our cohort, 78.7% had none of the 10 selected chronic diseases in 2014, 15.8% had only one chronic disease, 4.0% had two chronic diseases, and 1.5% had at least three chronic diseases. Among persons with two chronic diseases or less, the most frequent type of comorbidity was diabetes associated with respiratory illness (Table 4).

**Table 4:** Prevalence of comorbidities among the 10 selected chronic diseases (%, *n* = 469,255)^a^

	No other disease^b^	Stroke	Heart disease	Cancer^c^	Diabetes	Chronic kidney disease	Respiratory illness^d^	Cirrhosis	Alcohol use disorders	Major depression
No disease	79.88									
Stroke	0.39									
Heart disease	1.62	0.05								
Cancers^c^	1.58	0.02	0.08							
Diabetes	4.15	0.08	0.60	0.21						
Chronic kidney disease	0.32	0.01	0.07	0.04	0.11					
Respiratory illness^d^	5.34	0.05	0.33	0.23	0.66	0.06				
Cirrhosis	0.28	0.00	0.02	0.03	0.09	0.01	0.05			
Alcohol use disorders	0.61	0.02	0.04	0.02	0.06	0.01	0.11	0.10		
Major depression	1.22	0.01	0.04	0.05	0.12	0.01	0.19	0.01	0.06	
Neurological disorders	0.55	0.04	0.07	0.04	0.11	0.04	0.07	0.01	0.02	0.02

^a^Calculated among persons with two chronic diseases or less (98.5% of the sample)

^b^Of the nine other selected chronic diseases

^c^Breast/Lung/Colorectal/Stomach/Liver/Kidney/Pancreatic/Oesophageal

^d^Chronic obstructive pulmonary disease, asthma

### Per capita healthcare expenditure

For our cohort, the average healthcare expenditure per capita in 2014 was estimated at 2684 € (±7646 €). For those under 50 years old, costs were significantly higher among women (1841 vs. 1186 €; *P* < 0.001). However, for those aged 50 years and above, costs were not significantly different between men and women (4011 vs. 4029 €; *P* = 0.657).

### Cost associated with each chronic disease for people without comorbidity

Two-stage model estimations are provided in Table 5, as defined in the methods section by the quantity $\overline{\widehat{\mathit{\cos}{t}_{d_j}}}$, with the mean of each substrata-estimated marginal difference in the predicted outcome with the chronic disease variables d_j_ switched on or off. Column 1 of Table 5 indicates the figures and CIs among people with no comorbidity (involving the selected chronic diseases). The average estimated cost per capita associated with one chronic disease was the highest for CKD (8323 €, 95% CI 7090–9555 €) and the lowest for respiratory illnesses (1285 €, 95% CI 1103–1466 €). Please note that these figures relate to the weighted average estimates of the costs in 2014 for prevalent cases (detected before July 2013) and incident cases (diagnosed between July 2013 and June 2014). The method also allowed the generation of COI calculations in 2014, stratified by dates of diagnosis (results available on request).

**Table 5:** Average costs per capita in 2014 and 95% CI associated with the 10 selected chronic diseases according to type of comorbidity – two-stage model estimates (n = 476,252)

**Cost associated with…**		**Comorbidity**				
	**No other comorbidity**	**Stroke**	**Heart disease**	**Cancers** ^**a**^	**Diabetes**	**Chronic kidney disease**
Stroke	3466; 2973–3959		4203; 3524–4882	2784; 1913–3656	5659; 4804–6513	7833; 6032–9635
Heart disease	1828; 1570–2087	2566; 2055–3077		2156; 1721–2591	2687; 2293–3081	5172; 4215–6130
Cancers^a^	5115; 4322–5909	4434; 3229–5639	5443; 3229–5639		6240; 5143–7336	14,042; 11,528–16,556
Diabetes	1776; 1510–2041	3969; 3326–4611	2634; 2218–3051	2900; 2378–3423		8349; 7036–9662
Chronic kidney disease	8323; 7090–9555	12,690; 10,557–14,823	11,666; 9826–13,507	17,249; 14,365–20,134	14,895; 12,638–17,153	
Respiratory illness^b^	1285; 1103–1466	1775; 1374–2175	2332; 1956–2709	3016; 2258–3774	1955; 1663–2246	4440; 3630–5249
Cirrhosis	4225; 3623–4827	13,625; 10,987–16,263	5808; 4845–6771	12,402; 10,044–14,759	5377; 4543–6211	15,256; 12,593–17,920
Major depression	1528; 1303–1754	2421; 1356–3486	1929; 1527–2331	2803; 2020–3587	1879; 1565–2194	2900; 1664–4135
Neurological disorders	2121; 1798–2445	1617; 839–2395	2404; 1809–3000	3648; 2754–4541	3847; 3045–4649	1142; 239–2523
Alcohol user disorders	2323; 1924–2722	4303; 2939–5667	3319; 2712–3927	5492; 3820–7163	4056; 3401–4711	9344; 7457–11,231
**Cost associated with…**	**Comorbidity**					
	**Respiratory illness** ^**b**^	**Cirrhosis**	**Major depression**	**Neurological disorders**	**Alcohol use disorders**	
Stroke	3955; 3327–4584	12,865; 10,309–15,422	4358; 3136–5580	2961; 2200–3722	5445; 4167–6724	
Heart disease	2876; 2436–3316	3411; 2676–4146	2229; 1812–2645	2111; 1605–2618	2825; 2299–3350	
Cancers^a^	6847; 5508–8185	13,292; 10,624–15,960	6390; 5184–7596	6642; 5368–7915	8284; 6319–10,249	
Diabetes	2446; 2092–2800	2927; 2339–3516	2127; 1736–2517	3502; 2852–4151	3509; 2911–4107	
Chronic kidney disease	11477; 9762–13,193	19,354; 16,269–22,439	9694; 7992–11,396	7343; 5447–9240	15,344; 12,813–17,874	
Respiratory illness^b^		2331; 1837–2825	1389; 1003–1774	2275; 1866–2683	2180; 1810–2550	
Cirrhosis	1632; 1268–1997		5500; 4434–6565	6375; 5125–7626	4520; 3798–5242	
Major depression	1632; 1267–1998	2803; 1902–3703		2322; 1080–3565	1871; 1391–2350	
Neurological disorders	3111; 2587–3636	4271; 3254–5288	2915; 1607–4224		2393; 1877–2909	
Alcohol user disorders	3219; 2666–3772	2618; 1964–3271	2665; 2012–3318	2594; 2059–3129		

^a^Breast/Lung/Colorectal/Stomach/Liver/Kidney/Pancreatic/Oesophageal

^b^Chronic obstructive pulmonary disease, asthma

Note: To account for the different sociodemographic structures (e.g. younger age profiles for people without comorbidity), the same set of weights is used to estimate the average cost of the disease for people with and without comorbidity

### Extra cost associated with each chronic disease in the presence of comorbidity

Disease by disease, extra costs due to the presence of a comorbidity estimate varied greatly depending on the nature of the chronic disease and the comorbidity (Table 5, columns 2–11, see also Additional file 1 for the calculation method). The extra costs associated with diabetes were estimated at 1776 € without comorbidity, 2446 € (+670 €) when associated with respiratory illness, and 2634 € (+858 €) when associated with heart disease. The costs associated with heart disease were estimated at approximately 1828 € for individuals with no comorbidity, 2687 € (+859 €) for individuals with diabetes as comorbidity, 2566 € (+738 €) for individuals with stroke as comorbidity, and 2876 € (+1048 €) for individuals with respiratory illness as comorbidity.

The resulting events of super-additivity were assessed by the comparison described in inequality (3). A synthetic view is given in Table 6 for the 45 two-by-two possible comorbidities. There were only four cases where the costs were not super-additive.

**Table 6:** Assessment of ‘super-additivity’ for the 45 two-by-two possible comorbidity combinations (*n* = 476,252)

	Alcohol use disorders	Neurological disorders	Major depression	Cirrhosis	Respiratory illness^b^	Chronic kidney disease	Diabetes	Cancers^a^	Heart disease
Stroke	+	–	+	+	+	+	+	–	+
Heart disease	+	+	+	+	+	+	+	+	
Cancers^a^	+	+	+	+	+	+	+		
Diabetes	+	+	+	+	+	+			
Chronic kidney disease	+	–	+	+	+				
Respiratory illness^b^	+	+	+	–					
Cirrhosis	+	+	+						
Major depression	+	+							
Neurological disorders	+								

Note: The precise assessment of ‘super-additivity’ was computed on the basis of the comparison: $\overline{\widehat{\mathit{\cos}{t}_{d_{j\mid }{d}_k}}}+\overline{\widehat{\mathit{\cos}{t}_{d_{k\mid }{d}_j}}}<>\overline{\widehat{\mathit{\cos}{t}_{d_j}}}+\overline{\widehat{\mathit{\cos}{t}_{d_k}}}$ (3)

+ Left-hand side of inequality (3) is superior to the right-hand side, the assessment is ‘super-additivity’

– No super additivity (left-hand side of inequality (3) lower to the right-hand side)

^a^Breast/Lung/Colorectal/Stomach/Liver/Kidney/Pancreatic/Oesophageal

^b^Chronic obstructive pulmonary disease, asthma

### Aggregate costs

When these per capita costs were aggregated at national level, the chronic diseases with the highest estimated costs were cancer (6.8 billion €) followed by CKD and diabetes (6.4 billion €; Table 7).

**Table 7:** Aggregate costs in 2014 associated with the 10 selected chronic diseases (*n* = 476,252)

	Aggregate costs (real data, including the extra cost of comorbidity) (€)	Aggregate costs without diabetes^c^ (€)
Stroke	1,766,157,759	1,668,987,170
Heart disease	3,810,314,586	3,640,257,499
Cancers^a^	6,760,703,703	6,721,315,309
Diabetes	6,361,926,072	
Chronic kidney disease	6,349,510,855	5,868,318,845
Respiratory illness^b^	4,884,750,608	4,720,450,554
Cirrhosis	2,288,778,628	2,254,496,590
Major depression	1,345,807,466	1,326,877,079
Neurological disorders	1,439,130,111	1,345,681,049
Alcohol use disorders	1,550,325,340	1,508,304,205

^a^Breast/Lung/Colorectal/Stomach/Liver/Kidney/Pancreatic/Oesophageal

^b^Chronic obstructive pulmonary disease, asthma

^c^Simulation exercise in the absence of diabetes

Note: Stroke costs the French government 1766 million €. In the virtual exercise, where diabetes would be cured from society, stroke would only cost 1668 million €, because those patients suffering from stroke and diabetes would no longer have to bear the extra costs of stroke due to diabetes

### Simulation exercise without diabetes

When diabetes was ‘virtually’ cancelled from society (all sick people are cured of diabetes), we first saved 6.4 billion € as a direct effect (Table 7). However, diabetes may also interact super-additively with other treatment costs and, therefore, its cancelation would also have indirect effects; for example, the aggregate costs of CKD were significantly lower (−481 million €; Table 6) when diabetes was removed from the model. Termination of diabetes had also an impact on the aggregate cost of heart disease (−170 million €; Table 7), which is frequently associated with diabetes. A lesser impact was observed on the aggregate costs of respiratory illness (−164 million €) or stroke (−97 million €).

## Discussion

### Summary of results

The costs of the 10 selected chronic diseases were substantially higher for individuals with comorbidity (compared to similar agents without comorbidity), demonstrating the pattern of super-additive costs in cases of diseases interaction. Super-additivity was demonstrated for 41 cases out of the 45 couples studied. We also simulated preventive action on diabetes (prevalence set at 0%). Our results show that the system can save a significant amount of money not only on diabetes itself, but also on the chronic diseases that are frequently associated with diabetes. We estimate that the direct effect of diabetes disappearing is a saving of 6 billion €, but that the indirect effect is a saving of more than 1 billion €, cutting costs by an extra 18%. This points to severe underestimation of the economic benefits (returns) of preventive action, and confirms that comorbidity(ies) should be taken into account in COI analyses.

### Strengths and limitations

A major strength of the study is the estimation of disease-related healthcare costs both in the absence and presence of comorbidity. As recommended elsewhere [31], we used a regression-based bottom-up approach to estimate disease costs. Moreover, our COI analysis was performed on a very large sample of claim data. Compared to a top-down (attributable fraction) approach, estimates based on a bottom-up approach are more accurate because they can account for the occurrence of higher treatment intensity among those with the disease and, subsequently, the extra-expenditures for comorbidities are better captured [9, 31]; therefore, we were able to obtain more reliable estimates [26, 32]. By adding two-way interactions between the chronic disease variables in the two-stage cost model, we estimated the costs of multiple combinations of disease, and thus the extra cost of comorbidity. This enabled us to achieve accurate cost estimations and to precisely compute the super-additive monetary impact of comorbidity. To our knowledge, this kind of approach has not been used previously in a COI study.

Nevertheless, the study has several limitations. Firstly, drug-based diagnoses are only proxies for medical diagnoses. Using drug prescription data to identify individuals with chronic diseases could result in errors in prevalence/incidence estimates. However, in our view, the other methods used to detect illnesses (hospital discharge data and LTI) compensate for this.

Secondly, we limited our study to a selection of 10 chronic diseases. Since we examined a relatively low number of diseases, there are likely important comorbidities that have been missed. Including too few comorbidities in a cost regression model may lead to an overestimation of the effects of the comorbidities that are included if they are correlated with omitted comorbidities [33]. In order to limit this effect, we added the ‘other LTI’ variable in the two-step regression model, controlling for the presence of a chronic disease outside of the list of the 10 selected chronic diseases, and reducing unobserved heterogeneity.

Thirdly, this study was performed on a medico-administrative database from France, whose healthcare system comprises a fully integrated network of public hospitals, private hospitals, doctors and other medical service providers. The individual receiving care is generally reimbursed for all medical treatment on the basis of a price established by the Social Security administration, unlike practices in other countries. Access to care in France is very easy, with a large decisional autonomy allowed to both the physician and the patient, who are free to adapt treatments as they wish [34]. Therefore, a major limitation of our results regarding super-additivity concerns the generalisability of the results to other countries with different healthcare systems, relying, for instance, on tighter treatment protocols.

Fourthly, in the simulation exercise (where we eliminated diabetes), our estimate of savings only took into account the nine other comorbidities and their resulting super-additive costs. Further analyses should be performed to estimate how much more could be saved if a wider range of chronic and non-chronic diseases were considered, particularly comorbidities that are clinically related to the disease of interest [33].

Finally, the number of variables was very limited in our study (age, sex and estimated date of diagnosis) since we had no information on income level, educational level, employment status, supplementary mutual health insurance or the presence of risk factors (like tobacco/alcohol consumption, body mass index).

### Comparison with other COI studies

The literature offers competing estimates of disease costs, with each study having its own data and methods [2–7]. First, we found per capita healthcare expenditures similar to those estimated in 2010 by the French Directorate for Research, Studies, Assessment and Statistics [35] (2698 € vs. 2684 € in our study). In a French study published in 2003 [36], the total amount spent by the general health scheme on care for diabetic patients was estimated at 6 billion € in 2000, with 2.4 billion € attributed to the treatment of diabetes alone. In another study on the French ENTRED survey [37], including 6710 diabetic patients covered by the National Health Insurance, the total reimbursement cost for patients was estimated at 12.5 billion € in 2007, but the amount attributable to diabetes alone was not estimated. In a report published in 2007 by the French National Cancer Institute [38], cancer-related healthcare costs in 2004 were estimated at 11 billion €. However, in a study published in 2013 [39], cancer-related healthcare costs for France were estimated at 7 billion € in 2008.

## Conclusions

The main lessons from this paper are, firstly, that there are indeed considerable comorbidities in the French population – of the 21.3% of the population who suffer from the 10 selected illnesses, 25.7% are in fact suffering from more than one. Secondly, the treatment costs of the illnesses are clearly super-additive when they co-exist within the same patient, creating an extra cost that is ignored when disease treatments are considered separately. Our simulation exercise, although unrealistic, highlights this last point – if a disease like diabetes were to be avoided, the healthcare system could save not only the direct costs of diabetes, but also the extra costs that diabetes may generate through its interaction with other diseases. This represents more than 15% of the cost-of-diabetes valuation (and billions of euros), and suggests that the potential benefits of any preventive action against this kind of chronic disease are generally underestimated.

## Additional file

Additional file 1: Estimating the cost of chronic diseases in the presence of a comorbidity. (PDF 354 kb)

## Abbreviations

ATC: Anatomical Therapeutic Chemical
CI: confidence interval
CKD: chronic kidney disease
COI: cost of illness
ICD: International Classification of Disease
LTI: long-term illnesses
