# Supplementary material for: The extra cost of comorbidity: multiple illnesses and the economic burden of non-communicable diseases
Source: BMC Med. 2017 Dec 8;15:216. doi: 10.1186/s12916-017-0978-2 (PMC5723100; doi:10.1186/s12916-017-0978-2)
Supplement: Additional file 1: — Estimating the cost of chronic diseases in the presence of a comorbidity. (PDF 354 kb) [file 12916_2017_978_MOESM1_ESM.pdf]

Additional file 1. Estimating the cost of chronic diseases in the presence of a comorbidity

Reminder (equation 1). In each stratum (combination of age categories and gender), the increase in healthcare expenditure attributable to each disease was calculated by subtracting average predicted expenditure for sick people in each category, from average predicted expenditure for the individuals with the other disease variables set to 0 (or 1 in case of comorbidity for a given other disease). For example, in given strata i, the cost associated with chronic disease d_j (with no other comorbidity) is estimated as follows:

$$\widehat{cost_{\imath d_J}} = \left[\hat{c}_{i|d_j=1, d_{k_{k\neq j}}=0,\ d_{kl_{k,l\neq j}}=0*0, d_{jk_{k\neq j}}=1*0}\right] - \left[\hat{c}_{i|d_j=0, d_{k_{k\neq j}}=0,\ d_{kl_{k,l\neq j}}=0*0, d_{jk_{k\neq j}}=0*0}\right] \qquad (1)$$

with:

$\widehat{cost_{\imath d_J}}$: cost in 2014 associated with chronic disease j in strata i (no other comorbidity)

$\hat{c}_i$: predicted outcome in strata i

d_i: binary chronic disease variable for disease i

d_{jk} : interaction variable between chronic disease variables d_j and dj

In the presence of a comorbidity, the cost associated with chronic disease d_j (with disease d_k as only comorbidity) is estimated as follows:

$$\widehat{\mathrm{cost}_{\imath \mathrm{d_j}}}_{|\mathrm{d_k}} = \left[\hat{c}_{i|d_j=1,\ d_k=1, d_{l_{l\neq\{j,k\}}}=0,\ d_{jk}=1*1, d_{jl_{l\neq\{j,k\}}}=1*0, d_{kl_{l\neq\{j,k\}}}=1*0\ ,\ d_{lm_{l,m\neq\{j,k\}}}=0*0}\right] -$$

$$\left[\hat{c}_{i|d_j=0,\ d_k=1, d_{l_{l\neq\{j,k\}}}=0,\ d_{jk}=0*1, d_{jl_{l\neq\{j,k\}}}=0*0,\ d_{kl_{l\neq\{j,k\}}}=1*0\ ,\ d_{lm_{l,m\neq\{j,k\}}}=0*0}\right] \qquad (1\ \text{bis})$$

We obtained an average cost per capita attributable to each chronic disease by calculating a weighted mean over all strata:

$$\overline{\widehat{cost_{d_J}}} = \frac{1}{N}\sum_{i=1}^{N} w_i * \widehat{cost_{\imath d_J}} \qquad (2)$$

$$\overline{\widehat{cost_{d_J|d_k}}} = \frac{1}{N}\sum_{i=1}^{N} w_i * \widehat{\mathrm{cost}_{\imath \mathrm{d_j}}}_{|\mathrm{d_k}} \qquad (2\ \text{bis})$$

with:

$\overline{\widehat{cost_{d_J}}}$: COI calculation for disease d_j (no comorbidity)

$\overline{\widehat{cost_{d_J|d_k}}}$: COI calculation for disease d_j (among persons with disease d_k as comorbidity)

N: total number of strata (=combination of gender, age groups)

w_i: weight for stratum i (=number of individuals in the sample of persons with at least one disease)
